# Evaluating methamphetamine use and risks of injection initiation among street youth: the ARYS study

**DOI:** 10.1186/1477-7517-3-18

**Published:** 2006-05-24

**Authors:** Evan Wood, Jo-Anne Stoltz, Julio SG Montaner, Thomas Kerr

**Affiliations:** 1 British Columbia Centre for Excellence in HIV/AIDS, St. Paul's Hospital, 608 - 1081 Burrard Street, Vancouver BC V6Z 1Y6, Canada; 2 Department of Medicine, University of British Columbia, 3300 - 950 West 10th Avenue, Vancouver BC V5Z 4E3, Canada

## Abstract

Many Canadian cities are experiencing ongoing infectious disease and overdose epidemics among injection drug users (IDU). These health concerns have recently been exacerbated by the increasing availability and use of methamphetamine. The challenges of reducing health-related harms among IDU have led to an increased recognition that strategies to prevent initiation into injection drug use must receive renewed focus. In an effort to better explore the factors that may protect against or facilitate entry into injection drug use, the At Risk Youth Study (ARYS) has recently been initiated in Vancouver, Canada. The local setting is unique due to the significant infrastructure that has been put in place to reduce HIV transmission among active IDU. The ARYS study will seek to examine the impact of these programs, if any, on non-injection drug users. In addition, Vancouver has been the site of widespread use of methamphetamine in general and has seen a substantial increase in the use of crystal methamphetamine among street youth. Hence, the ARYS cohort is well positioned to examine the harms associated with methamphetamine use, including its potential role in facilitating initiation into injection drug use. This paper provides some background on the epidemiology of illicit drug use among street youth in North America and outlines the methodology of ARYS, a prospective cohort study of street youth in Vancouver, Canada.

## Background

It is estimated that approximately 340,000 Americans [1] and 100,000 Canadians are current injection drug users (IDU) [2]. Injection drug use can lead to overdose, infectious disease, loss of social and economic functioning and extensive engagement in criminal activity. In addition to the morbidity and mortality associated with infectious diseases [3], overdose fatalities (usually opioid) among IDU have been a leading cause of death within the general population in many urban areas in North America in recent years [4,5], including British Columbia, Canada, where approximately one overdose death per day was recorded throughout the late 1990s [6]. At a societal level, injection drug use has created public health and fiscal crises, with multiple costs to public health care and auxiliary services as well as the welfare and criminal systems [7-9]. Costs associated with treatment of human immunodeficiency virus (HIV) and hepatitis C virus (HCV) infection are also high [10]. In recent years, injection drug use has been estimated to account for approximately 25% of new HIV infections and 63% of new HCV infections in Canada, with similar rates observed in the US [11,12]. In Vancouver, an explosive outbreak of HIV infection was documented among IDU during the mid- to late-1990s, characterized by an HIV incidence rate of 18 per 100 person-years in 1997, one of the highest rates ever reported in the developed world [13].

Recent reports of increasing injection drug use and high-risk behavior by street youth in North America highlight the growing risk of HIV transmission among younger age groups and the urgent need to evaluate and inform primary prevention strategies among this population [14]. Street youth are particularly vulnerable to experiencing health-related harms for a variety of reasons. These include: lack of education about drug use, sexual health risks; sexual and physical violence; poverty and neglect; and precarious living conditions, either on the street or in risky relationships, or both [15,16]. Not surprisingly, injection drug use has become a growing problem among this population. In the US, estimates of the prevalence of injection drug use among street youth range from 30% to 40% [17-19], while a national study from the US found approximately 28% of street youth and a further 10% of youths living in shelters had participated in prostitution [20]. Estimates of the number of street youth in Canada have ranged as high as 150,000 [21], with injection drug use reported by 38% and 54% of these individuals in Vancouver [22] and Montreal [23], respectively. As a consequence, street youth are increasingly recognized as being among the highest at risk of those sub-populations at risk for HIV and HCV infection.

Studies of factors associated with initiation of injection drug use have suggested that close friends introduce the majority of both males and females to injection drug use [24,25]. The mean age of individuals initiating injection ranges from 16 to 18 years, and the "introducer" is typically an IDU several years older [24-26]. Roy *et al*. found that among Montreal street youth, more girls than boys required assistance injecting and were less likely to use a clean needle. Further, although a high proportion of new initiates (84%) did inject with a clean needle, only 62% used clean drug preparation equipment [23]. More recently, prospective studies of youth transitioning from non-injection drug use to injection drug use have identified a number of independent predictors for initiation of injection [15,27,28]. Fuller *et al*. identified race other than African American, exclusive crack smoking just prior to initiating, smoking marijuana, high school dropout, and sex trading during the year prior to transition, particularly among young females, as correlates of transition from non-injection to injection drug use in a cohort of young high-risk drug users in Baltimore [27,28]. Roy *et al*. have reported from a cohort study in Montreal that: having been homeless; being under 18 years of age; being tattooed; recent use of heroin, hallucinogens, cocaine, crack, or freebase; being female with an IDU friend; and having ever experienced extra-familial sexual abuse were all associated with initiation of injection drug use by street youth [15].

Nevertheless, large numbers of street youth exhibit many of the above risk factors and do not transition into injection drug use [22,29]. As such, many questions remain with regard to why some drug users transition into injection drug use and some do not [30]. In particular, there is uncertainty regarding other factors that may facilitate initiation into injection drug use, such as the precise roles of injection drug users in one's social network [31,32]. The role of expanded access to syringes, in settings where syringe exchange has become decentralized, has also not been explored.

Although it is well documented that the environment, social networks, health policy, and accessibility of interventions may contribute to or diminish the risk of HIV and HCV infection [10,13,33,34], published data about macro-level risk factors associated with initiation of drug injection are scarce. Vancouver has recently initiated several secondary prevention programs aimed at reducing HIV incidence rates, including a large decentralized needle distribution program with a flexible exchange policy and two medically supervised injecting clinics, yet no programs are in place to evaluate the impact of these policies on street youth [35]. In addition, in recent years, evidence has suggested that the use of methamphetamine has grown in western Canada, with Vancouver being the primary site of this increase. Not surprisingly, methamphetamine is commonly used by Vancouver street youth, and the health-related harms associated with this practice remain under-investigated.

In light of the above concerns, and the fact that infectious diseases and other harms persist despite HIV prevention programming targeted towards injection drug users, it has recently been argued that the injection-related infection risk hierarchy should be updated so that the prevention of injection drug use is given greater priority [31,32]. Consistent with this call, a cohort of street youth has been initiated in Vancouver, Canada. Known as the At Risk Youth Study (ARYS, pronounced 'arise'), the study will seek to examine the impact of the local decentralized syringe distribution scheme and supervised injecting sites on the rates of initiation of injection drug use. The working hypothesis is that knowledge of and exposure to harm reduction programs among non-injecting youth will not be associated with increased rates of initiation into injection drug use. The ARYS study will also seek to examine the impact of methamphetamine use on various health-related harms, as well as the potential role of methamphetamine use in transitioning into injection drug use. In this case, the working hypothesis is that smoked methamphetamine will be associated with subsequent initiation into injection drug use. In keeping with recent developments related to improving reporting quality of non-randomized evaluations of behavioral and public health interventions [36], this paper describes the methodology being employed in the ARYS study to investigate risk factors for initiation into injection drug use as well as potential health-related harms of methamphetamine use.

## Methods

### Recruitment and follow-up

The recruitment strategy for ARYS involves standard techniques for reaching hidden populations, and recruitment will be conducted from the city's streets and from youth agencies and services [37-39]. Since there are no registries from which to draw street youth, the sample can be viewed as a convenience sample, although major efforts are being undertaken to try to maximize the representativeness of the sample. This includes extensive street-based outreach, including outreach during the nighttime, and efforts to have street youth recruit their peers. Outreach has also been systematically undertaken in a range of neighborhoods around the city where street youth are known to congregate.

After initial contact is made, the nature of the study is explained and informed consent is offered to those who wish to enroll. Although these recruitment techniques are inferior to random recruitment methods, random recruitment of street youth was viewed to be impractical in our setting, and we are unaware of any large prospective study of street youth that has employed these methods. Eligibility criteria include age (14 to 26 years) and use of drugs other than marijuana in the past 30 days. Eligibility is not restricted to those youth who have already begun injecting, and although ORALscreen drug test kits are being used to assess illicit drug use levels at baseline, this screen will not be used to exclude potential enrollees.

Data collection procedures for the ARYS cohort are similar to other prospective cohort studies of illicit drug users whereby individuals provide a baseline blood sample for measurement of HIV and hepatitis C (HCV) antibodies and complete an interviewer-administered questionnaire. Pre- and post-test counseling and referral to health services are provided as part of the study. To enable high rates of follow-up, contact information is obtained and individuals are requested to return to the study site every six months for the duration of the study, at which time blood is again sampled for evaluation of HIV and HCV incidence and a detailed follow-up questionnaire is administered. A five-dollar incentive is also offered to youth to return after three months to check in and update their contact information, and thus far the majority of youth have checked in at the three-month mark. In addition, the vast majority of youth who have enrolled to date have provided email addresses for follow-up purposes.

Although it was expected at the outset that follow-up with this particular population would be challenging, it is anticipated that these strategies may prove invaluable in ensuring high rates of follow-up despite the issues of mobility common among street youth.

### Outcome ascertainment

Outcome ascertainment for the ARYS cohort will involve blood testing, clinical evaluation of needle tracks, and self-reported behavioral data obtained through the interviewer-administered questionnaire. In addition, the local setting is unique because of the availability of confidential record linkages made possible through Canada's universal healthcare system. Specifically, administrative databases create an opportunity for the accurate ascertainment of key measures, including emergency room and hospital use, medication use, and contact with various harm reduction services, including the city's supervised injecting facility. These linkages have several advantages, since self-reported health service use has been shown to be subject to socially desirable responding [40].

Although many youth-specific indicators have had to be developed, the survey instrument for the ARYS cohort is largely based on the scales that have been developed as part of the Vancouver Injection Drug Users Study (VIDUS), a prospective cohort study of IDU that has been described in detail previously [13,41-43]. The survey instruments have been intentionally coordinated to facilitate the examination of the natural history of injection drug use through to adulthood. Both surveys include sections on sources of income, non-injection and injection drug use (including overdose and binging), interactions with police, incarceration, sexual activity, drug and alcohol treatment, violence, and nutritional needs. Both surveys also include standardized measures for depression (Centre for Epidemiologic Studies Depression Scale [44]) and childhood trauma (Childhood Trauma Questionnaire [45,46]), as well as HIV knowledge scales [47] and a non-standardized self-efficacy scale to evaluate self-efficacy to avoid injection drug use. The youth survey also includes sections on educational background and exposure to injection drug use. The coordination of survey instruments allows us to seek to explore the relationship between established injectors and new initiates into injection drug use.

### Additional data sources

The above prospective cohort data will be augmented by a number of other data sources. First, quantitative activities of the ARYS cohort will be informed by a newly developed qualitative research program which will involve in-depth qualitative interviews with street youth to further explore areas of interest. For instance, street youth who transition into injection drug use during follow-up will be targeted for qualitative interviews so that the circumstances of initiation into injection drug use can be further explored. Second, there will also be an active ethnographic research team who will undertake field observations of drug use behaviors among street youth in natural settings. When making observations and conducting unstructured interviews in natural settings, study staff will use a verbal script to inform potential participants about the research, its purpose, and the risks involved. We will also obtain verbal informed consent before observing and recording data in the natural settings of parks, streets, and alleyways where drug consumption activities are occurring and where street youth congregate. In instances where street youth express that they are not willing to be observed or participate, the researcher will remove him/herself from the immediate vicinity and attempt to engage with street youth in another locale. Individuals participating in unstructured qualitative interviews go through a similar informed consent process before interviews are undertaken and field notes are recorded.

## Results and discussion

Enrollment into ARYS began in October 2005, and approximately 324 youth have been recruited to date. Preliminary evaluation of the cohort shows that participants are approximately 72% male, 25% Aboriginal, and the median age is 22 (inter-quartile range is 21–24). Notably, 50% of all participants report either currently being or having been injection drug users.

Unique ethical issues relating to methodology arise when working with a cohort that includes legal minors. Because one of the objectives of the study is to expand our current understanding of the relationship between childhood sexual and physical abuse and initiation of injection drug use, questions of that nature are included on the questionnaire. However, because of the legal duty to report abuse of persons under the age of 19 in our jurisdiction (British Columbia), researchers are placed in the position of having to carry out that legal duty in the course of collecting these data from minors. This limitation to confidentiality is spelled out in the consent form, and participants are assured that they can refuse to answer questions about abuse (or any other topic) if they choose. When participants under age 19 choose to disclose abuse in the course of answering survey questions, interviewers are trained to follow up on the duty to report. All efforts are made to report with the participant's consent and full knowledge, and participants are offered referrals to free and available community counseling services. Questions about abuse are situated within the nurse's questionnaire, as it was felt that participants would be more comfortable disclosing and participating in reporting the abuse with an experienced community health nurse. Further, two of the study's investigators hold doctoral degrees in counseling psychology and are available for clinical supervision when disclosure of abuse occurs. Extensive consultation with the government ministry responsible for investigating reports of abuse occurred before the study commenced, as well as with the chair of the research ethics board prior to submitting the application for ethics approval of the study. The challenge in terms of methodology is to collect these important data from participants within a research protocol that satisfies the legal duty to report abuse of minors and ensures a standard of care in the process.

## Conclusion

In summary, the recent reports of increasing injection drug use and high-risk behavior by street youth in North America highlight the growing risk of HIV transmission among younger age groups and the urgent need to evaluate and inform primary prevention strategies within this population [14]. At present, many questions remain with regard to why some drug users transition into injection drug use and some do not [30], and it has recently been argued that the injection-related infection risk hierarchy should be updated so that the importance of prevention of injection drug use is emphasized [31,32].

Vancouver, Canada, has recently initiated several secondary prevention programs, including a large decentralized needle distribution program as well as two medically supervised injecting clinics, aimed at reducing HIV incidence rates among active injection drug users [35]. In addition, the city has experienced a substantial increase in methamphetamine use among street youth. In response to the above issues, the ARYS cohort has been developed to examine risk factors for initiation into injection drug use and the harms of methamphetamine use among street youth in this environment. Using the methodology described above, recruitment was initiated in October 2005, and initial reports from the ARYS cohort are expected in the summer of 2006.

## Competing interests

The author(s) declare that they have no competing interests.

## Authors' contributions

EW, JS, TK drafted the manuscript and addressed the reviewers' helpful suggestions. All authors participated in the drafting of the manuscript and approved the final version.
